# Metal-on-Metal Total Hip Arthroplasty Associated with Adverse Local Tissue Reaction and Pseudoaneurysm of the Superior Gluteal Artery: A Case Report and Literature Review

**DOI:** 10.1055/s-0036-1593855

**Published:** 2016-10-31

**Authors:** Amit Shah, Rajesh Botchu, David Dunlop, A. Mark Davies, Steven L. James

**Affiliations:** 1 Department of Musculoskeletal Radiology, Royal Orthopaedic Hospital, Birmingham, United Kingdom; 2 Department of Orthopaedics, Royal Orthopaedic Hospital, Birmingham, United Kingdom

**Keywords:** metal-on-metal hip arthroplasty, adverse local tissue reaction, pseudotumor, pseudoaneurysm, superior gluteal artery

## Abstract

Adverse local tissue reaction (ALTR) and pseudoaneurysm formation are rare but known complications following metal-on-metal hip total hip arthroplasty (THA). We report the first known case in the English literature of a concurrent unilateral ALTR and pseudoaneurysm of the superior gluteal artery in the same patient. Following minimal rise in serum metal ions, an ultrasound of the right hip demonstrated an avascular solid/cystic lesion anterolaterally in keeping with an ALTR. More posterolaterally, a second discrete thick-walled cystic lesion was identified. Doppler interrogation demonstrated a “yin yang” pattern suggestive of a pseudoaneurysm. Magnetic resonance imaging confirmed the presence of an anterolateral periarticular lesion with a second discrete lesion within the gluteus medius. Subsequent computed tomography angiography confirmed the presence of arterial contrast blush within the posterior gluteal lesion adjacent to the superior gluteal artery. The patient remains asymptomatic and is being managed conservatively. We review the imaging characteristics of ALTR and pseudoaneurysm occurring post-THA. When a complex solid/cystic lesion is encountered in a patient with a THA, radiologists must ensure that the lesion is interrogated with color Doppler to confidently distinguish a pseudotumor from a pseudoaneurysm. This information is vital to the surgeon to avoid unexpected hemorrhage if revision joint replacement surgery is being contemplated.

Hip arthroplasty is a common procedure, with nearly 70,000 operations in the United Kingdom in 2014. 1 Traditional metal-on-polyethylene (MoP) components generate particulate debris, leading to an inflammatory response and resulting in osteolysis and aseptic loosening. To overcome this, metal-on-metal (MoM) devices have increased in popularity, especially in young patients, with the aim of potentially improving wear rates compared with MoP implants. 2 There are several potential complications including postoperative joint effusion, iliopsoas bursitis, tendinitis or impingement, and gluteal tendinopathy. Two rare complications include the formation of a pseudotumor or adverse local tissue reaction (ALTR) and pseudoaneurysm.

We present a case of an asymptomatic patient with a MoM total hip arthroplasty (THA) with an ALTR and pseudoaneurysm of the superior gluteal artery found incidentally during ultrasound assessment for raised serum metal ions. To the best of our knowledge, this is the first reported case in the English literature of two rare post-THA complications occurring unilaterally and concurrently in the same patient.

## Case Report

**Fig. 1:**
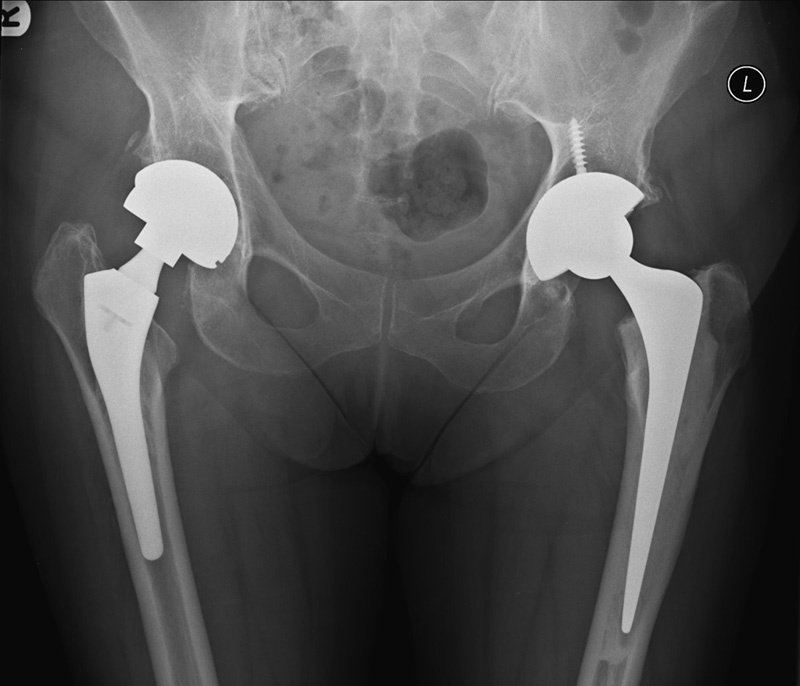
Anteroposterior radiograph demonstrating bilateral total hip arthroplasties with no adverse features.

**Fig. 2:**
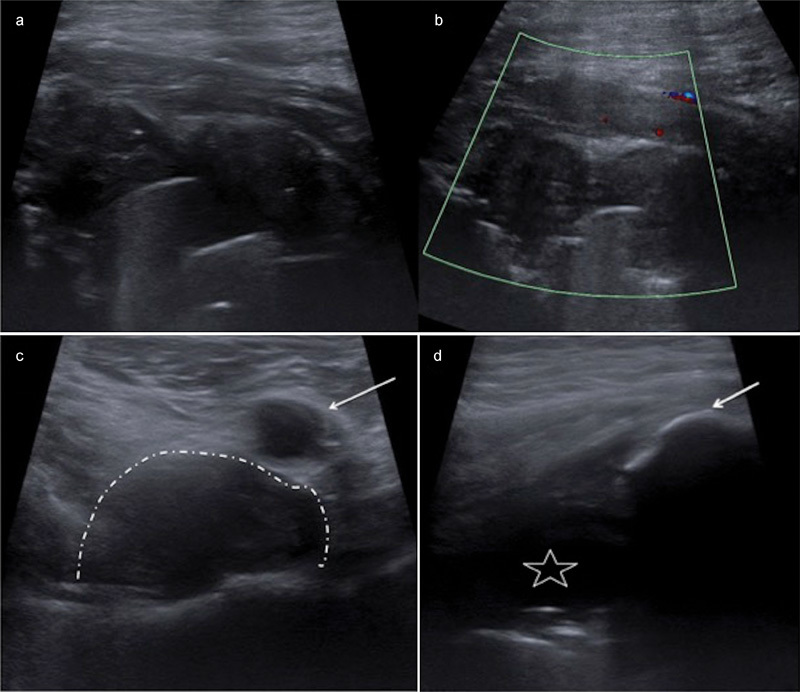
Longitudinal ultrasound image (a) of the anterior right hip joint demonstrating a mixed echogenic solid/cystic mass. Longitudinal ultrasound Doppler image (b) confirms a lack of vascularity. The transverse ultrasound image (c) confirms extensions of this fluid lesion into the iliopsoas bursa (dotted curve). The white arrow indicates the common femoral artery. Longitudinal ultrasound image (d) demonstrates fluid collection (white star) extension laterally (white arrow indicates the greater trochanter). Appearances are in keeping with an adverse local tissue reaction in a patient with a metal-on-metal hip arthroplasty and raised serum metal ions.

**Fig. 3:**
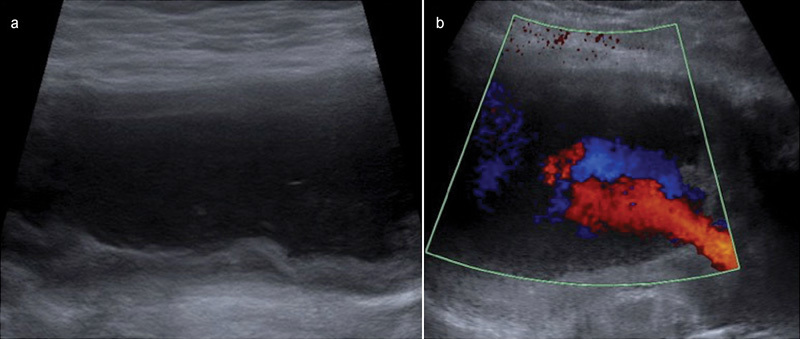
Transverse ultrasound image more posterolaterally (a) demonstrates a second thick-walled cystic structure. Transverse Doppler image (b) demonstrates turbulent blood flow producing a yin yang pattern characteristic of a pseudoaneurysm.

**Fig. 4:**
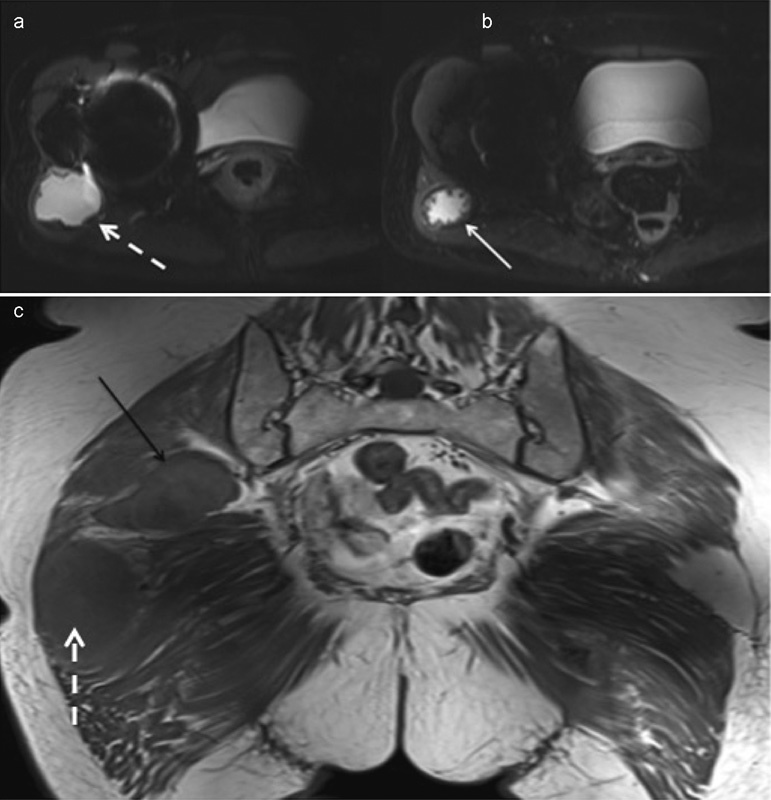
Axial short tau inversion recovery magnetic resonance imaging (MRI) confirms a periarticular cystic structure related to the right hip joint (dashed white arrow in a and c) with a low signal pseudocapsule (solid white arrow in b) in keeping with an adverse local tissue reaction (ALTR). The ALTR (dashed white arrow) is isointense to skeletal muscle on coronal spin-echo T1 sequence (c). There is a second discrete lesion (solid black arrow in c) within the gluteal muscle with a rim that is isointense to muscle and intralesional hypointensity suggestive of turbulent flow in keeping with a pseudoaneurysm.

**Fig. 5:**
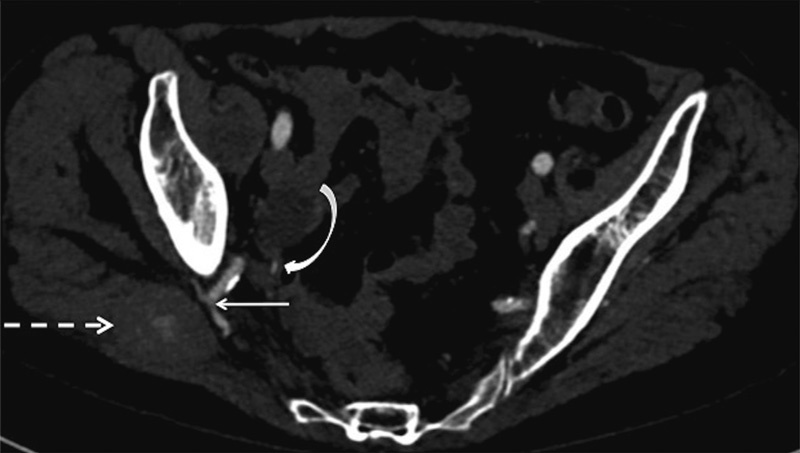
Axial computed tomography angiogram demonstrates enhancement of the superior gluteal artery (straight white solid arrow), which is adjacent to a fusiform mass (dotted white arrow) within the gluteal muscles. The mass demonstrates internal contrast blush in keeping with a pseudoaneurysm. The curved white arrow indicates the inferior gluteal artery.

A 71-year-old woman underwent a left MoM Birmingham hip resurface (BHR) procedure in 2005 and subsequently had a MoM THA on the right in 2008. The left BHR was revised to a MoM THA in 2012 after worsening of left hip pain. She was monitored annually with serum metal ions and hip radiographs. Her baseline serum cobalt was 158 nmol/L and serum chromium was 119 nmol/L. The Medicines and Healthcare Products Regulatory Agency reference threshold for serum chromium was 134 nmol/L and for serum cobalt was 119 nmol/L. Because she was asymptomatic, a clinical decision was made to monitor serum metals ion levels and to obtain imaging if levels remained high. During clinical review in 2014, the patient reported very occasional discomfort on the right, after strenuous activity, and remained asymptomatic on the left. Clinical examination was unremarkable. Repeat serum ion levels rose slightly, and therefore a radiograph and an ultrasound were requested to assess the right hip. The radiograph demonstrated bilateral THAs in situ but was otherwise unremarkable ( Fig. 1 ). The ultrasound demonstrated a large joint effusion with synovitis and an anterior periarticular cystic lesion with no internal vascularity ( Fig. 2a and b ). The collection extended into the iliopsoas bursa ( Fig. 2c and d ). Further posterolaterally, there was a thick-walled fluid collection ( Fig. 3a ), separate to the anterolateral collection. Doppler interrogation revealed internal flow in a “yin yang” pattern, highly suggestive of a pseudoaneurysm ( Fig. 3b ). Magnetic resonance imaging (MRI) confirmed the large ALTR related to the THA and a second discrete gluteal fusiform soft tissue mass ( Fig. 4 ). The lesion demonstrated hypointense signal internally, on all sequences, suggestive of turbulent flow. Subsequent computed tomography (CT) angiography confirmed arterial contrast blush of the fusiform mass in the right buttock in keeping with a pseudoaneurysm ( Fig. 5 ). A neck to its parent vessel could not be identified but was in the territory of the superior gluteal artery. The patient has elected for conservative management because she remains asymptomatic and is being clinically followed.

## Discussion

### ALTR/Pseudotumor

Although the wear rate is lower with MoM devices, 3 MoM prostheses release metal nanoparticles and ions into the body. 4 5 The resultant inflammatory response leads to the development of a benign sterile inflammatory soft tissue mass. Particulate debris is detrimental to the durability of MoP implants; so too is an ALTR, which can have drastic effects on the MoM implant and make revision surgery difficult. The reported incidence of ALTR following MoM THA is between 0 and 6.5%. 6 *Pseudotumors* , *metal hypersensitivity* , *aseptic lymphocyte-dominated vasculitis-associated lesions* , *adverse local tissue reactions* , and *adverse reaction to metal debris* are all terms that have been used to describe this rare complication. 7

The commonest ions released from MoM alloy implants are cobalt (Co) and chromium (Cr). In 2010, the UK Medicines and Healthcare Products Regulatory Agency released a Medical Device Alert in response to the increasing number of revision surgeries for ALTR. 8 Recommendations included regular clinical follow-up, investigation of those patients with painful MoM arthroplasties, and regular measurement of serum Co and Cr ion levels. Imaging was emphasized for those patients with two separate serum metal ion levels above serum Cr of 134 nmol/L and Co of 119 nmol/L. Of note, Fang and coworkers concluded that serum metal ion measurements were not on their own sufficiently accurate for monitoring the presence of a pseudotumor in asymptomatic patients. 9 Thus, patients need to be assessed clinically, biochemically, and radiologically to identify an ALTR.

ALTR can present months to years after surgery, is commoner in females and in patients under 40 years of age, and usually involves both hips in the case of bilateral THAs. 10 Patients can present with hip pain, spontaneous dislocation, neurologic symptoms, or palpable masses. 11 It is well recognized that an ALTR can be identified in asymptomatic patients on ultrasound and MRI, as was seen in our patient. 12 The pathogenesis of an ALTR is unclear but is initiated by the release of metal ions due to excessive wear of the cobalt-chrome prosthesis leading to an inflammatory response. Two distinct histopathologic observations have been made, one of predominant macrophage infiltration with little necrosis and one of a predominantly lymphocyte-mediated response with necrosis termed *aseptic lymphocyte-dominated vasculitis-associated lesion* , 13 14 15 16 which is thought to represent a delayed type IV hypersensitivity reaction. 15

Radiographs of ALTR are usually normal ( Fig. 1 ) and are performed to assess the prosthesis and exclude loosening or periprosthetic fracture. A subtle but recognizable radiographic finding is resorption of the calcar femorale, but this finding does not reflect extensive soft tissue changes. 17 ALTRs are better appreciated on ultrasound, CT, and MRI, although metal artifacts degrade the latter two imaging methods. On ultrasound, the anterior, lateral, and posterior aspects of the joint should be interrogated carefully. ALTR occurs more often in posterior or lateral locations followed by anterior, which is typically related to the iliopsoas bursa. 18 This corresponds to the commonly used surgical approaches. 19 Interestingly, a pseudotumor has been reported in the urinary bladder, thought to be secondary to cement extrusion through acetabular defects. 20 On ultrasound, ALTR may appear as an anechoic or hypoechoic fluid collection with little vascularity, which may demonstrate thick echogenic septations and marked synovial hypertrophy ( Fig. 2a ). 18 ALTRs are intimately related to the joint capsule and communicate with the hip joint ( Fig. 4a ). 9 ALTR appears on CT as a solid or cystic periarticular mass. CT is also useful in assessing periprosthetic bony changes. 21

Numerous studies have demonstrated the usefulness of MRI in detecting an ALTR. Basic sequences consist of axial T1-weighted and T2-weighted fast spin-echo and coronal T1-weighted sequences and short-tau inversion recovery sequences. Metallic artifact can be “directed” to optimize the region of interest by alternating phase and frequency encoding directions. Furthermore, metal artifact reduction sequences have made possible the use of MRI in patients with THAs. Metal artifact reduction sequences MRI was found to be superior to CT for the diagnosis and characterization of ALTR. 21 MRI appearances suggestive of an ALTR consist of periprosthetic collections originating from the neck of the femoral component to the surrounding soft tissues. These are typically isointense to muscle on T1-weighted images ( Fig. 4c ). On T2-weighted images, the fluid is usually hyperintense with a thick irregular pseudocapsule ( Fig. 4b ). The pseudocapsule is low signal intensity on T1-weighted and T2-weighted imaging caused by susceptibility artifact from the nanoparticles in the pseudocapsule. 9 17 21 22

Management of pseudotumors is complex, and revision surgery may increase the risk of complications. Surgery tends to be more complex secondary to the soft tissue destruction. 23 Outcomes for revision surgery for ALTR/pseudotumors are considerably worse than the outcome of other THA. 24

### Pseudoaneurysm

Vascular injuries, such as pseudoaneurysm formation, are a rare complication after THA. 25 However, their potential must be considered due to the proximity of the vascular structures to the hip joint. 26 Proposed intraoperative mechanisms include retractor injury, thermal injury from methyl methacrylate, or direct penetration from polymer or gouging during acetabular preparation. Postoperative mechanisms include acetabular component or cement migration leading to vascular erosion. 27 The incidence of all vascular injuries post-THA is estimated between 0.08 and 0.67%, with an average of 0.2 to 0.3%. 28 Vascular injuries have been reported with both anterolateral or posterior surgical approaches with pseudoaneurysm formation of the profunda femoris and medial circumflex arteries. 29 30

Gluteal artery pseudoaneurysms represent less than 1% of all aneurysms with the majority of reported cases secondary to blunt or penetrating trauma or iatrogenic injury during injection or biopsy or due to acetabular component migration. 31 32 The superior gluteal artery is more commonly injured than the inferior gluteal artery. 33

Presentation with a tender mass, which may be pulsatile, may occur weeks or months following THA. Other symptoms reported include hypotension, shock, limb edema, sciatic nerve palsy, and a bleeding wound fistula. 26 Our case is unusual as the pseudoaneurysm was asymptomatic and found incidentally post-THA.

Imaging findings of a pseudoaneurysm include demonstration of a lesion contiguous with the parent artery with a patent or thrombosed lumen neck. Doppler ultrasound is the key to distinguish an ALTR from a pseudoaneurysm; a pseudoaneurysm will reveal arterial flow. The turbulent forward and backward flow often produces the characteristic yin yang sign on color flow ( Fig. 3b ) and a to-and-fro pattern with pulsed Doppler imaging. 34 35 Unenhanced CT may demonstrate a low-attenuation lesion with a neck arising from the parent artery. Attenuation within the pseudoaneurysm will depend on the composition of acute and chronic blood products. A CT angiogram will demonstrate contrast blush into the pseudoaneurysm ( Fig. 5 ). A communication with a parent artery may be visible, but like in our case cannot also be readily identified if the pseudoaneurysm arises from a small branch vessel. On MRI, arterial flow can manifest as a signal flow void on a noncontrast study with avid enhancement following administration of gadolinium. Concentric rings of thrombus in differing stages of evolution create heterogenous signal intensities on T1- and T2-weighted MRI, with a characteristic “blooming” artifact of hemosiderin on gradient-echo sequences as a result of increased magnetic susceptibility. 36 Pulsation artifact may also be identified on MRI.

Several treatment strategies for pseudoaneurysm have been proposed, including direct suture, closure of the defect with a patch, resection of the aneurysm, thromboembolization, and endovascular repair.

## Conclusion

THA is the second most common joint replacement surgery, 1 and although complications of ALTR and pseudoaneurysm formation are rare entities on their own, we have presented a case where both have occurred unilaterally and concurrently. When a complex solid/cystic lesion is encountered in a patient with a THA, radiologists must ensure that the lesion is interrogated with color Doppler to confidently distinguish a pseudotumor from a pseudoaneurysm. This information is vital to the surgeon to avoid unexpected hemorrhage if revision joint replacement surgery is being contemplated.
